# Age‐Stratified Performance of the TAPSE/sPAP Ratio as a Marker of Right Ventricular‐Pulmonary Arterial Coupling in Chronic Kidney Disease

**DOI:** 10.1111/echo.70461

**Published:** 2026-04-17

**Authors:** Görkem Yıldız, Tolga Kunak

**Affiliations:** ^1^ Department of Cardiology Faculty of Medicine Yüksek İhtisas University Ankara Türkiye; ^2^ Department of Cardiology Faculty of Medicine Akdeniz University Antalya Türkiye

**Keywords:** age stratification, chronic kidney disease, echocardiography, right ventricular function, TAPSE/sPAP ratio

## Abstract

**Background:**

Right ventricular (RV) dysfunction in chronic kidney disease (CKD) remains underrecognized. The tricuspid annular plane systolic excursion to systolic pulmonary artery pressure ratio (TAPSE/sPAP) reflects RV‐pulmonary arterial coupling, but its relationship with CKD severity is unknown. To evaluate the association between TAPSE/sPAP ratio and CKD stage severity and assess age‐related effect modification.

**Methods:**

This cross‐sectional study enrolled 120 participants: 40 patients with stage I–II CKD, 40 with stage III–IV CKD, and 40 healthy controls. All underwent echocardiography and nephrologist evaluation. Proportional odds regression models adjusted for cardiovascular risk factors evaluated the TAPSE/sPAP‐CKD association. ROC analysis assessed discriminatory performance. Age‐stratified analyses (<65 vs. ≥65 years) evaluated effect modification.

**Results:**

TAPSE/sPAP ratio declined progressively across CKD stages (controls: 0.67 ± 0.11, stage I–II: 0.61 ± 0.13, stage III–IV: 0.53 ± 0.13 mm/mmHg; *p *< 0.01). Each 0.10‐unit decrease was independently associated with advanced CKD (adjusted OR 2.08, 95% CI 1.49–2.89, *p* < 0.001). TAPSE/sPAP ratio achieved the highest AUC (0.734, 95% CI 0.625–0.833), outperforming TAPSE alone (AUC 0.615; DeLong *p *= 0.049), while statistically comparable to sPAP (AUC 0.715; DeLong *p *= 0.400). Age‐stratified analysis revealed excellent performance in patients <65 years (AUC 0.819, sensitivity 78.6%, specificity 86.8%) but limited utility in those ≥65 years (AUC 0.579; *p *= 0.015). Random forest analysis identified age (35.8%) and TAPSE/sPAP (22.0%) as dominant predictors.

**Conclusions:**

TAPSE/sPAP ratio is an independent marker of CKD severity enabling non‐invasive detection of RV‐PA uncoupling, with excellent discriminatory performance in younger but limited utility in older patients, suggesting that age‐specific interpretation and integration with renal biomarkers are recommended.

## Graphical abstract

The echocardiographic TAPSE/sPAP ratio independently predicts advanced CKD stage (adjusted OR 2.08 per 0.10‐unit decrease; bootstrap AUC 0.735). Performance is excellent in patients younger than 65 years (AUC 0.819, sensitivity 78.6%, and specificity 86.8%) but attenuated in older adults (AUC 0.579), supporting age‐stratified thresholds for cardiorenal risk stratification.

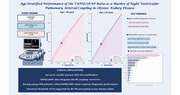

## 1 Introduction

Chronic kidney disease (CKD) is defined as functional or structural impairment of the kidneys that persists for at least three months [1]. The prevalence of CKD in the world population is 8%–16%, and it is the 16th leading cause of death worldwide [2]. Higher levels of cardiovascular and total mortality begin below an estimated glomerular filtration rate (eGFR) of 75 mL/min/1.73 m^2^ and increase with continued decline in eGFR. The highest mortality is reached when eGFR falls below 15 [3].

Cardiorenal syndrome (CRS) is a disease process in which cardiac and renal dysfunction coexist. Cardiovascular diseases that develop in patients with CKD are examined in the CRS class IV (renocardiac syndrome) subgroup [4]. Many studies have shown that left ventricular (LV) hypertrophy, systolic dysfunction, and dilation of the left ventricle and left atrium develop within the backdrop of CKD [5, 6]. However, changes in the right heart in CRS are less well known. Pulmonary hypertension (PHT), dilatation of the right ventricle and right atrium, and a decrease in tricuspid annular plane systolic excursion (TAPSE) are known changes in the right heart [7]. The ratio of TAPSE to systolic pulmonary arterial pressure (TAPSE/sPAP) is a noninvasive measure of right ventricle–pulmonary artery (RV–PA) connectivity that indicates the adaptation of right ventricular (RV) contractility to afterload. Therefore, it is a reliable and noninvasive method for direct evaluation of right heart hemodynamics [8]. Moreover, no studies were found in the literature evaluating the RV–PA connection with the TAPSE/sPAP ratio for different CKD stages. In light of this information, this study was designed to analyze the relationship between the stages of CKD and RV hemodynamics based on TAPSE/sPAP ratio.

## 2 Materials and Methods

### 2.1 Study Design and Population

This single‐center, cross‐sectional, observational study was approved by the local ethics committee (protocol 2024/10/23, dated February 19, 2025). As a non‐interventional observational study, this research was not registered in a clinical trials registry. Following written informed consent, patients aged 18–85 years with CKD were prospectively enrolled from the Nephrology Outpatient Clinics of Yüksek İhtisas University between March and August 2025. All participants were evaluated by a nephrologist including medical history, anthropometric measurements, physical examination, and laboratory tests. Based on CKD staging, 40 patients with stage I–II formed Group 1, 40 patients with stage III–IV formed Group 2, and 40 healthy volunteers formed the control group. Accordingly, this cohort was evaluated in the context of CKD‐related cardiac involvement, consistent with type 4 cardiorenal syndrome (renocardiac syndrome), rather than cardiorenal syndrome type 2. Exclusion criteria included: stage V CKD, acute kidney injury, renal transplant, pulmonary thromboembolism (PTE), chronic obstructive pulmonary disease (COPD), heart failure, primary pulmonary hypertension, dialysis, left ventricular ejection fraction (LVEF) <50%, severe valvular disease, inadequate echocardiographic image quality, and unmeasurable sPAP (Figure 3).

We hypothesized that the TAPSE/sPAP ratio would decline with CKD progression. The sample size was calculated at 95% confidence and 80% power assuming that TAPSE/sPAP would be >0.55 mm/mmHg in controls, 0.32–0.55 mm/mmHg in Group 1, and <0.33 mm/mmHg in Group 2 (SD = 0.15), based on established thresholds for pulmonary hypertension (0.55 mm/mmHg) and high mortality risk (≤0.32 mm/mmHg) [9].

### 2.2 Chronic Renal Failure

The medical history, physical examination, anthropometric measurements, and laboratory values for participants were initially evaluated by a nephrologist. Each person's eGFR was calculated using the chronic kidney disease epidemiology collaboration equation (CKD‐EPI 2021), where S_Cr_ is standardized serum creatinine in mg/dL, *κ* is 0.7 for women and 0.9 for men, α is –0.241 for women and –0.302 for men, and min(S_Kr_/*κ*,1) is minimum S_Cr_ /*κ* or 1’i. Age (in years) is included in the equation, and the final unit for the eGFR equation is mL/min/1.73 m^2^.

$$\begin{array}{ccc} \mathrm{eGFR} & = & 142\times \min{\left(S_{\mathrm{Kr}}/\kappa \right)}^{\alpha }\times \max\left(S_{\mathrm{Kr}}/\kappa ,1\right)\\ & & \times \;0.9938^{\mathrm{age}}\left(\times \;1.012\;\mathrm{for}\;\mathrm{women}\right) \end{array}$$

CKD staging and patient selection criteria:

- **Group 1 (Stage I‐II CKD)**: eGFR ≥60 mL/min/1.73 m^2^ sustained for ≥3 months **plus evidence of kidney damage** (persistent albuminuria [urine albumin‐to‐creatinine ratio ≥30 mg/g], structural abnormalities on renal ultrasound, or known primary renal disease).
- **Group 2 (Stage III‐IV CKD)**: eGFR: 15–59 mL/min/1.73 m^2^ sustained for ≥3 months.
- **Control group**: eGFR ≥90 mL/min/1.73 m^2^ with no evidence of kidney damage (normal urinalysis, no albuminuria, normal renal ultrasound).

Patients with eGFR <15 mL/min/1.73 m^2^ (Stage V) or receiving renal replacement therapy were excluded.

### 2.3 Transthoracic Echocardiography

After patient recruitment and nephrologist evaluation, transthoracic echocardiography (TTE) (Affiniti CVx or EPIC S4‐1 probe, Phillips Healthcare Systems, Andover, MA, USA) was performed by a cardiologist unaware of the initial evaluation. All TTE examinations were performed according to guidelines [10, 11]. LVEF was calculated from two‐ and four‐chamber images using the Simpson biplane method. All valves were examined structurally and functionally. LV diastolic function was assessed based on the ratio of the transmitral peak E velocity to the mean septal and lateral mitral annular velocity (E/e′). The RV parameters of TAPSE, maximal tricuspid regurgitation velocity (TRVmax), estimated sPAP, and RV end‐diastolic diameter were measured. TAPSE was determined by measuring the total displacement in millimeters of the lateral tricuspid annulus in M‐mode recordings of end‐systole and end‐diastole on apical four‐cavity images. In patients with severe tricuspid insufficiency (TI), assessment of RV function with TAPSE is not accurate [12]. Therefore, patients with severe TI were excluded from the study. sPAP was calculated with the formula sPAP = 4(TRVmax)^2^ + SAB after determination of TRVmax and right atrial pressure (RAP). RAP was determined from the diameter of the inferior vena cava and its respiratory variation [10].

### 2.4 Statistical Analysis

SPSS 25.0 (SPSS Inc., Chicago, IL, USA) was used for statistics. Normally distributed data were expressed as mean ± SD, and nonnormally distributed data as median (interquartile range). The Mann–Whitney U test, Student *t* test, and chi‐square test were used for non‐normal, continuous, and categorical variables, respectively. ANOVA with post‐hoc tests compared variables across groups. ROC analysis evaluated predictive performance; Youden J statistic determined optimal thresholds. Pairwise AUC comparisons were performed using the DeLong method to evaluate statistical differences in discriminatory performance between, sPAP, TAPSE, and TAPSE/sPAP ratio in general and in age stratification.

Single and multiple ordinal logistic regression analyses evaluated independent predictors. Proportional odds (PO) and partial proportional odds (PPO) models were compared using likelihood ratio tests and AIC; the PO specification was retained when PPO did not improve fit.

To further assess the robustness of the findings and the relative importance of candidate variables, a random forest model was constructed using 1000 trees with 5‐fold cross‐validation. Variable importance was quantified by mean decrease in Gini index. Bootstrap internal validation with 1000 replications was additionally performed to estimate model optimism and assess overfitting. These analyses were intended as supportive internal validation of the primary statistical findings rather than as a standalone predictive machine learning model. All analyses were performed using Python 3.12 and scikit‐learn 1.3.0.

## 3 Results

A total of 120 patients were included in the study. Forty patients with stage I–II CKD were in group 1, 40 patients with stage III–IV CKD were in group 2, and 40 healthy individuals were in the control group. Group 1 consisted of 13 (32.5%) stage I and 27 (62.5%) stage II patients, and group 2 consisted of 23 (57.5%) stage III and 17 (42.5%) stage IV patients. The sex distributions of the control and study groups were similar (*p* < 0.01), with female *n* = 21 (52.5%), *n* = 25 (62.5%), and *n* = 17 (42.5%) for the control group, group 1, and group 2, respectively. While the age distributions of the control group and group 1 were similar (42.1 ± 11.9 years vs. 50.0 ± 18.51 years, respectively, *p* = 0.07), group 2 was significantly older than both the control group and group 1 at 64.9 ± 15 years versus 42.1 ± 11.9 years (*p* < 0.01) and 50.0 ± 18.51 (*p* < 0.01), respectively. Similarly, creatinine and eGFR values were significantly higher in group 2 compared to the other two groups (*p* < 0.01). Angiotensin receptor blocker (ARB) or angiotensin converting enzyme inhibitor (ACEI) use was significantly higher in group 2 than in the control group, with *n* = 20 (50%) versus *n* = 9 (23%), respectively (*p* = 0.03). Body mass index (BMI), smoking, presence of diabetes (DM), hypertension (HT), dyslipidemia, coronary artery disease (CAD) and use of beta‐blockers (BB), calcium channel blockers (CCB), and thiazide diuretics were similar among the groups (Table 1).

**TABLE 1:** Baseline characteristics and echocardiographic parameters of the control and study groups.

A. Baseline characteristics	Control group (*n* = 40)	Group 1 Stage I–II CKD (*n* = 40)	Group 2 Stage III–IV CKD (*n* = 40)	*p* value
Female, *n* (%)	21 (52.5%)	25 (62.5%)	17 (42.5%)	0.203
Age (mean years ± SD)	**42.1 ± 11.9^a^**	**50.0 ± 18.51^a^**	**64.9 ± 15.3^b^**	**<0.01**
BMI (mean kg/m^2^ ± SD)	28.96 ± 3.93	29.11 ± 4.31	28.04 ± 4.23	0.461
eGFR (mean mL/min/1.73 m^2^ ± SD)	**85.45 ± 11.68^a^**	**80.7 ± 12.63^a^**	**36.8 ± 12.60^b^**	**<0.01**
Chronic kidney disease				
Stage I, *n* (%)		13 (32.5%)		
Stage II, *n* (%)		27(62.5%)		
Stage III, *n* (%)			23 (57.5%)	
Stage IV, *n* (%)			17 (42.5%)	
Diabetes, *n* (%)	9 (23%)	12 (30%)	16 (40%)	0.236
Hypertension, *n* (%)	10 (25%)	15 (38%)	18 (45%)	0.169
Dyslipidemia, *n* (%)	9 (23%)	11 (28%)	17 (43%)	0.131
ACEI/ARB use, *n* (%)	9 (23%)^a^	18 (45%)^ab^	20 (50%)^b^	0.027
Thiazide use, *n* (%)	3 (7.5%)	4 (10%)	5 (12.5%)	0.757
B. Echocardiographic parameters				
LVEF (%, mean ± SD)	61.3 ± 5.3	60.9 ± .42	58.9 ± 5.6	0.134
Mitral E/e′ (mean ± SD)	8.39 ± 0.39	8.50 ± 1.29	8.89 ± 1.37	0.164
LV septum thickness (mm, mean ± SD)	9.9 ± 1.4	10.2 ± 2.0	10.8 ± 1.6	0.057
RV basal EDD (mm, mean ± SD)	34.5 ± 3.2	34.5 ± 3.4	35.2 ± 2.8	0.550
RA minor diameter (mm, mean ± SD)	32.7 ± 3.6	33.5 ± 4.1	34.5 ± 5.0	0.155
TAPSE (mm, mean ± SD)	**19.49 ± 1.77^a^**	**18.78 ± 1.56^ab^**	**18.26 ± 2.10^b^**	**0.013**
sPAP (mmHg, mean ± SD)	**29.8 ± 5.4^a^**	**32.0 ± 6.1^a^**	**36.2 ± 6.9^b^**	**<0.01**
TAPSE/sPAP(mm/mmHg, mean ± SD)	**0.67 ± 0.11^a^**	**0.61 ± 0.13^a^**	**0.53 ± 0.13^b^**	**<0.01**

*Note*: Groups with statistical differences in the ANOVA post‐hoc analyses are marked with different letters, and groups with similarities are marked with the same letter.

Abbreviations: ACEI, angiotensin converting enzyme inhibitor; ARB, angiotensin receptor blocker; CKD, chronic kidney disease; eGFR, estimated glomerular filtration rate; LA, left atrium; LVEF, left ventricular ejection fraction; RA, right atrium; RV, right ventricle; SD, standard deviation; sPAP, systolic pulmonary artery pressure; TAPSE, tricuspid annular plane systolic excursion.

Bold values are different between grups and the difference is statistically significant.

The TTE performed on the study and control groups showed that LVEF, left atrial (LA) anteroposterior (AP) diameter, basal right ventricular ejection fraction (RVEF), and right atrial (RA) minor diameter were similar among the groups. TAPSE was significantly lower in group 2 compared to the control group (18.26 ± 2.10 mm vs. 19.49 ± 1.77 mm, respectively, *p* = 0.009). Similarly, the TAPSE/sPAP ratio was also lower in group 2 compared to the other groups, at 0.53 ± 0.13 mm/mmHg compared to the control group at 0.67 ± 0.11 mm/mmHg and group 1 at 0.61 ± 0.13 mm/mmHg (*p* < 0.01). sPAP was significantly higher in group 2 (36.2 ± 6.9 mmHg) than in the control group (29.8 ± 5.4 mmHg) and group 1 (32.0 ± 6.1 mmHg) (*p* < 0.01). The post‐hoc analyses showed TAPSE, sPAP, and the TAPSE/sPAP ratio to be similar between the control group and group 1 (Table 1).

In the adjusted proportional odds model (age, sex, hypertension, diabetes, ejection fraction, coronary artery disease, hyperlipidemia, and septal thickness as covariates), a lower TAPSE/sPAP ratio was independently associated with more advanced CKD category (*β* = −7.34, *p* < 0.001). Expressed on a clinically interpretable scale, each 0.10‐unit decrease in TAPSE/sPAP was associated with higher odds of being in a more advanced CKD category (adjusted OR 2.08, 95% CI 1.49–2.89). Allowing a threshold‐specific (PPO) effect for TAPSE/sPAP did not improve model fit (likelihood ratio test *p* = 0.731; AIC 221.84 vs. 219.96), supporting the proportional odds specification for reporting.

In the adjusted proportional odds model including TAPSE and sPAP (with age, sex, hypertension, diabetes, ejection fraction, coronary artery disease, hyperlipidemia, and septal thickness as covariates), both lower TAPSE and higher sPAP were independently associated with a more advanced CKD category. Specifically, each 1‑mm decrease in TAPSE was associated with higher odds of being in a higher CKD stage (adjusted OR 1.45, 95% CI 1.15–1.82; *p* = 0.0016), and each 5‑mmHg increase in sPAP was associated with higher odds of being in a higher CKD stage (adjusted OR 1.82, 95% CI 1.30–2.55; *p* < 0.001). A partial proportional odds specification allowing threshold‐specific effects for TAPSE and sPAP did not improve model fit compared with the proportional odds model (likelihood ratio test *p* = 0.562; AIC 220.13 vs. 217.29); therefore, the proportional odds model was retained for reporting (Table 2).

**TABLE 2:** Multivariable proportional odds (ordinal logistic) regression models for groups.

Model	Parameters (scaled)	Adjusted OR (95% CI)	*p* value
Model A (ratio model)	**TAPSE/sPAP ratio (per 0.10 decrease)**	**2.08 (1.49–2.89)**	**<0.001**
Model B (TAPSE+ sPAP model)	**TAPSE (per 1 mm decrease)**	**1.45 (1.15–1.82)**	**0.002**
	**sPAP (per 5 mmHg increase)**	**1.82 (1.30–2.55)**	**<0.001**
	**Age (per 1‐year increase)**	**1.10 (1.06–1.14)**	**<0.001**
	Sex (male vs. female)	1.06 (0.41–2.74)	0.907
	Hypertension (yes vs. no)	1.39 (0.37–5.20)	0.624
	Diabetes (yes vs. no)	2.03 (0.85–4.84)	0.115
	EF (per 1% increase)	1.13 (1.03–1.24)	0.008^a^
	CAD (yes vs. no)	0.68 (0.24–1.95)	0.472
	Hyperlipidemia (yes vs. no)	0.62 (0.25–1.58)	0.305
	LV septum thickness (per 1 unit)	0.97 (0.66–1.41)	0.869

*Note*: Groups: 0 = Control/healthy, 1 (group 1) = patients with stage I–II chronic kidney disease (CKD), 2 (group 2) = patients with stage III–IV CKD. PPO (partial proportional odds) specifications allowing threshold‐specific effects did not improve model fit compared with the proportional odds specification in either model (Model A: LR *p* = 0.731; Model B: LR *p* = 0.562); therefore, proportional odds results are reported.

Abbreviations: CAD, coronary artery disease; EF, ejection fraction; LV, left ventricular; sPAP, systolic pulmonary artery pressure; TAPSE, tricuspid annular plane systolic excursion.

Bold values are statistically significant ones.

^a^A partial proportional odds (PPO) specification allowing threshold‐specific effects did not improve model fit compared with the proportional odds model (likelihood ratio tests *p* = 0.731 for the TAPSE/sPAP ratio model and *p* = 0.562 for the TAPSE+ sPAP model); therefore, proportional odds estimates are reported.

Although EF was statistically significant in the fully adjusted model, sensitivity analyses indicated that this association was not robust to removal of age from the model (EF became non‐significant and changed direction), suggesting that the EF coefficient may primarily reflect multivariable adjustment patterns related to the strong correlation between EF and age rather than an independent effect of EF on CKD stage.

**FIGURE 1:**
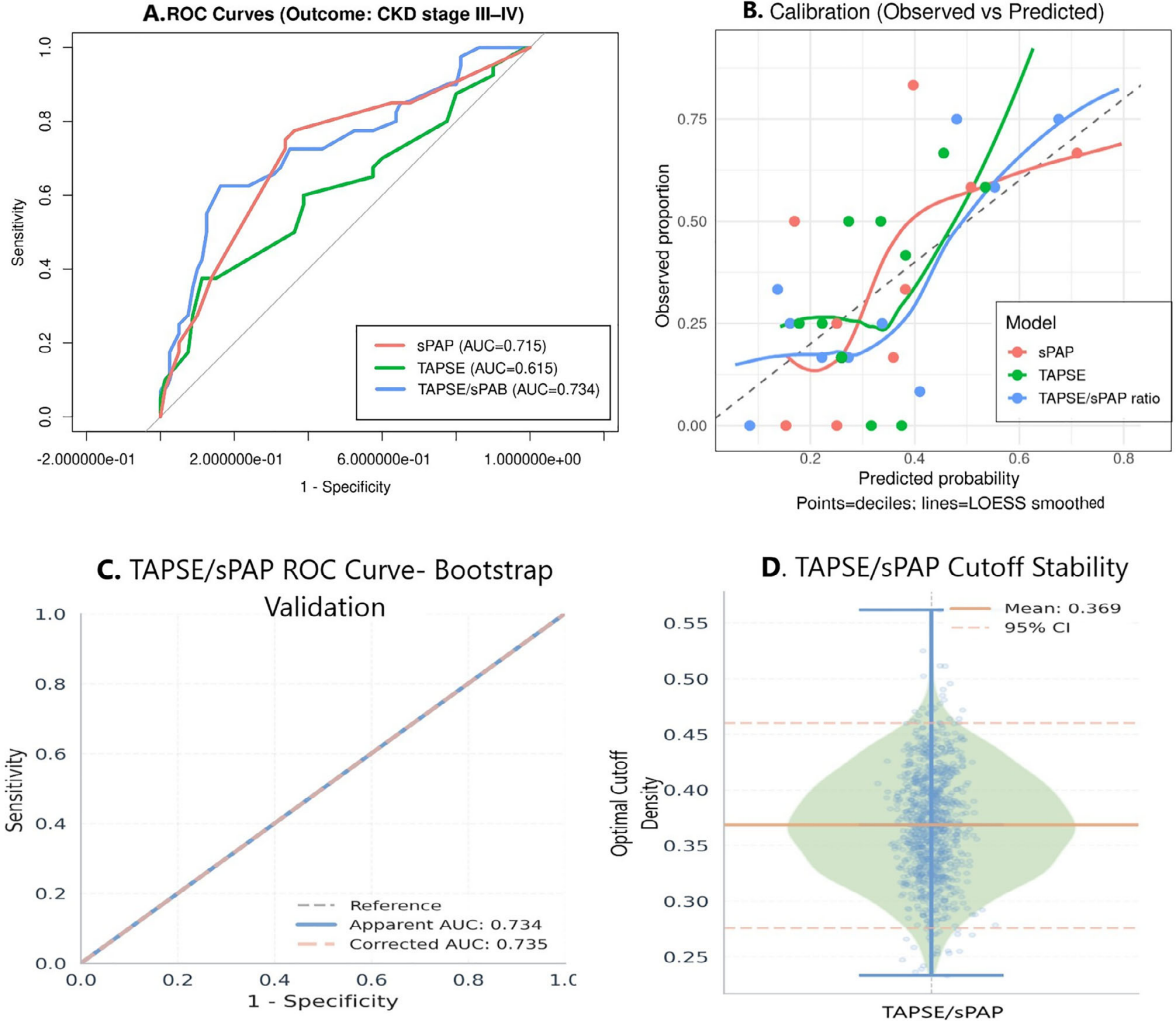
ROC analysis of TAPSE, sPAP, and TAPSE/sPAP ratio, calibration plots and bootstrap internal validation of TAPSE/sPAP ratio model. Outcome is stage III–‐IV chronic kidney disease (Group 2). (A ROC analysis for the discriminative power of TAPSE, sPAP, and TAPSE/sPAP ratio in predicting stage III–IV CKD: TAPSE (AUC: 0.615, 95% CI: 0.504–0.727, *p* = 0.040); sPAP (AUC = 0.715, 95% CI = 0.614–0.815, *p* < 0.01); TAPSE/sPAP ratio performed best (AUC = 0.734, 95% CI = 0.634–0.833, *p* < 0.01). (B) Calibration plots (observed vs. predicted) for identifying CKD stage III–IV (Group 2). Points represent deciles of predicted risk and curves are LOESS‐smoothed estimates; the dashed line indicates perfect calibration. Models are based on single‐predictor logistic regression using TAPSE, sPAP, and TAPSE/sPAP ratio. (C) TAPSE/sPAP ROC curve‐bootstrap validation show excellent concordance between apparent (AUC: 0.734) and corrected (AUC: 0.735) performance. (D) Optimal cutoff stability (mean: 0.369, 95% CI: 0.276–0.460) with minimal optimism (–0.0017) confirms robust performance without overfitting. AUC, area under the curve; CKD, chronic kidney disease; LOESS, locally estimated scatterplot smoothing; ROC, receiver operating characteristic curve; sPAP, systolic pulmonary arterial pressure; TAPSE, tricuspid annular plane systolic excursion.

In the ROC analysis performed to determine the predictive power of parameters for stage III–IV CKD, TAPSE had an AUC of 0.615 (95% CI = 0.504–0.727, *p* = 0.040), indicating statistically significant but limited diagnostic power. sPAP demonstrated high discrimination (AUC = 0.715, 95% CI = 0.614–0.815, *p* < 0.01), and the TAPSE/sPAP ratio achieved the highest AUC numerically (AUC = 0.734, 95% CI = 0.625–0.833, *p* < 0.01). DeLong pairwise comparisons revealed that TAPSE/sPAP ratio significantly outperformed TAPSE alone (*Z* = 1.967, *p* = 0.049), while its discriminatory performance was statistically comparable to sPAP (*Z* = 0.843, *p* = 0.400); sPAP and TAPSE also did not differ significantly (*Z* = 1.322, *p* = 0.186). The optimal cutoff for TAPSE/sPAP ratio was ≤0.52, yielding sensitivity of 62.5% and specificity of 83.7% (Figure 1A). Calibration analyses for identifying stage III–IV CKD demonstrated excellent agreement between predicted and observed risks across all three models: calibration‐in‐the‐large was near optimal (intercepts ∼0) and calibration slopes were 1.00 for all predictors, indicating no systematic over‐ or under‐estimation of risk. The TAPSE/sPAP ratio yielded the lowest Brier score (0.186), followed by sPAP (0.193) and TAPSE (0.210), consistent with its superior overall predictive accuracy (Figure 1B).

Bootstrap internal validation (1000 replications) demonstrated minimal overfitting (optimism‐corrected AUC 0.735 vs. apparent AUC 0.734; mean optimism –0.0017), confirming model robustness (Figure 1C,D).

Random forest analysis (1000 trees, 5‐fold CV; AUC: 0.861 ± 0.063) identified age as the strongest predictor (35.8%), followed by TAPSE/sPAP ratio (22.0%), sPAP (12.6%), and TAPSE (8.4%). Notably, TAPSE/sPAP ratio substantially outranked its individual components, confirming its superiority as an integrated marker.

Decision curve analysis demonstrated positive net benefit across clinically relevant thresholds (10%–50%), with the TAPSE/sPAP ratio model outperforming both “screen all” and “screen none” strategies, supporting its value for routine risk stratification.

### 3.1 Age‐Stratified Analysis

**FIGURE 2:**
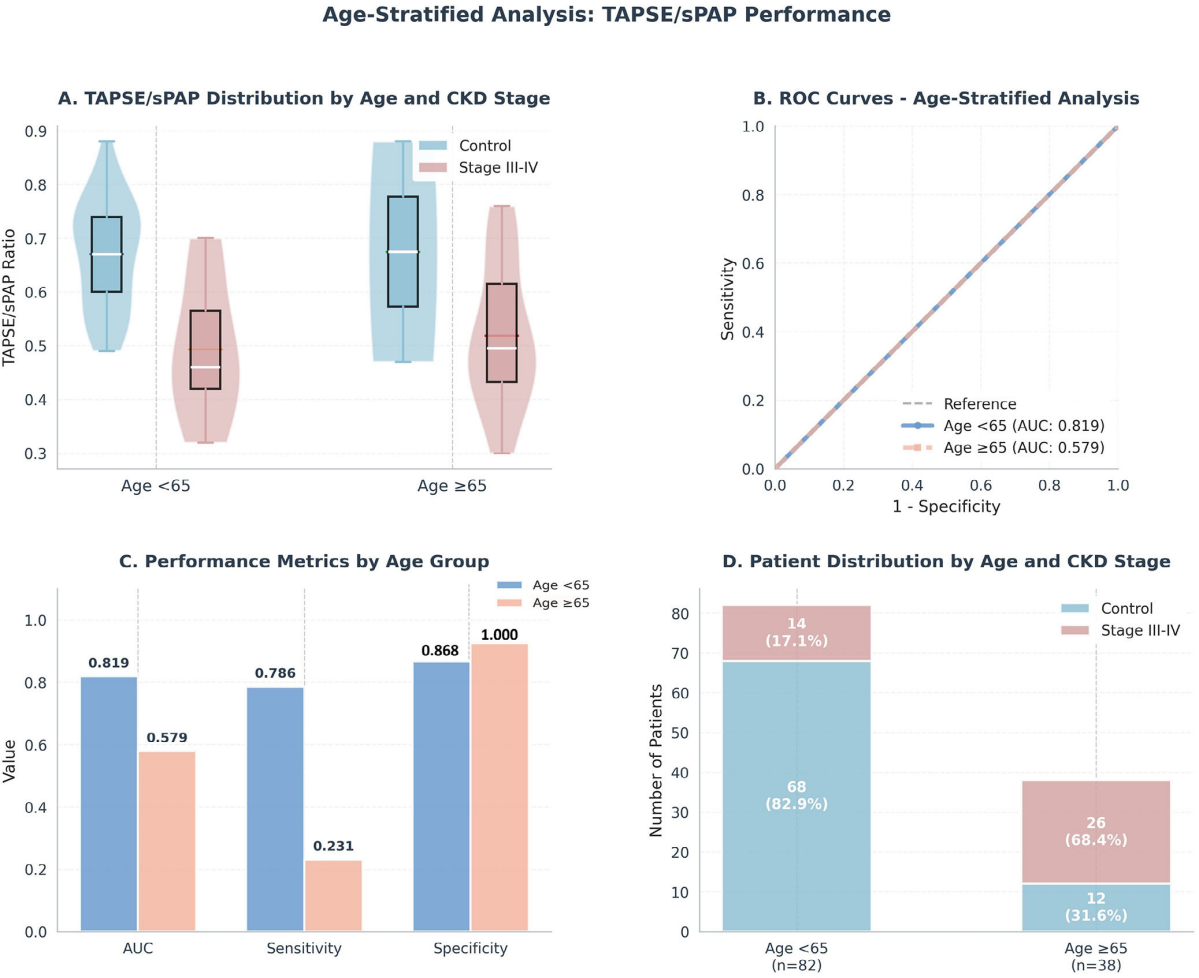
Age‐stratified analysis of TAPSE/sPAP performance. (A) Distribution of TAPSE/sPAP ratios by age group and CKD stage, showing violin and box plots. (B) ROC curves comparing discriminatory ability in younger (AUC: 0.819) versus older (AUC: 0.579) patients. (C) Performance metrics (AUC, sensitivity, specificity) stratified by age group, demonstrating superior performance in younger patients. (D) Patient distribution showing higher prevalence of advanced CKD in older group (68.4% vs. 17.1%). AUC, area under the curve; CKD, chronic kidney disease; ROC, receiver operating characteristic curve; sPAP, systolic pulmonary arterial pressure; TAPSE, tricuspid annular plane systolic excursion.

TAPSE/sPAP ratio demonstrated strong discriminative ability in patients <65 years (*n* = 82, AUC: 0.819, sensitivity: 78.6%, specificity: 86.8%) but limited performance in those ≥65 years (*n* = 38, AUC: 0.579). This age‐dependent difference likely reflects higher baseline prevalence of advanced CKD in older patients (68.4% vs. 17.1% Stage III–IV) and overlap in TAPSE/sPAP values between CKD stages (Figure 2).

**FIGURE 3:**
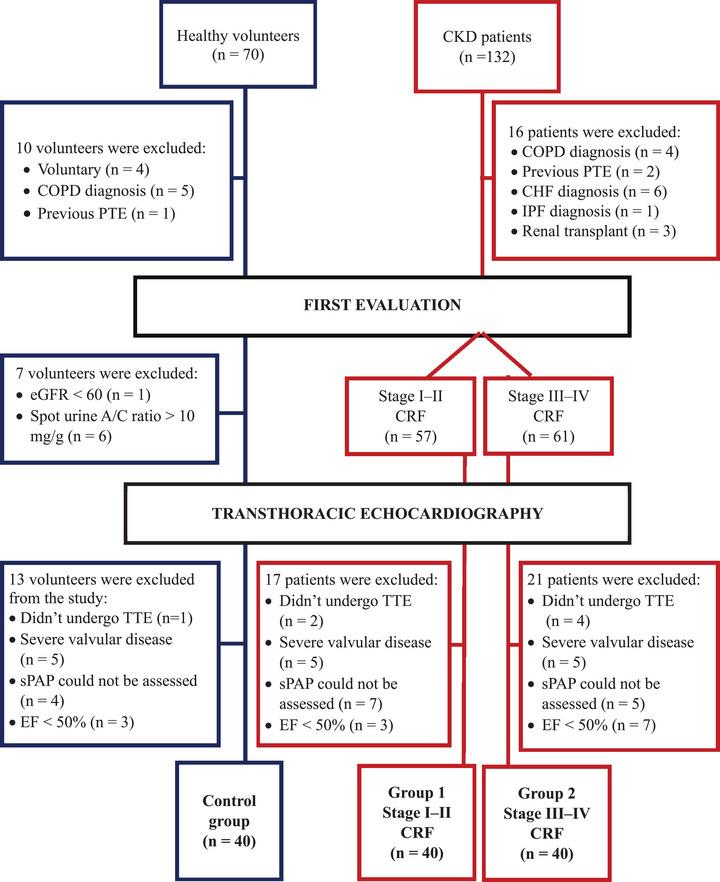
Flow chart illustrating participant enrollment, exclusion criteria, and final study groups. A total of 120 participants were enrolled following rigorous screening: 40 healthy controls, 40 patients with stage I–II chronic kidney disease (CKD), and 40 patients with stage III–IV CKD. Major exclusion criteria included severe valvular disease, reduced left ventricular ejection fraction (<50%), inability to assess systolic pulmonary artery pressure (sPAP), chronic obstructive pulmonary disease (COPD), congestive heart failure (CHF), previous pulmonary thromboembolism (PTE), idiopathic pulmonary fibrosis (IPF), and renal transplantation. All participants underwent transthoracic echocardiography (TTE) with a comprehensive assessment of right ventricular function and pulmonary hemodynamics. A/C, albumin‐to‐creatinine; CHF, congestive heart failure; CKD, chronic kidney disease; COPD, chronic obstructive pulmonary disease; EF, ejection fraction; eGFR, estimated glomerular filtration rate; IPF, idiopathic pulmonary fibrosis; PTE, pulmonary thromboembolism; sPAP, systolic pulmonary artery pressure; TTE, transthoracic echocardiography.

## 4 Discussion

This is, to our knowledge, the first systematic evaluation of RV–PA coupling across CKD stages using the TAPSE/sPAP ratio. CKD progression demonstrated a consistent hemodynamic pattern: sPAP increased (29.8→36.2 mmHg, *p* < 0.01) while TAPSE/sPAP declined (0.67→0.53 mm/mmHg, *p* < 0.01), indicating deteriorating RV‐PA coupling under rising afterload. In multivariable models adjusted for cardiovascular risk factors, TAPSE/sPAP was independently associated with more advanced CKD (OR 2.08 per 0.10‐unit decrease, 95% CI 1.49–2.89, *p* < 0.001) with better overall discriminative performance than its individual components (AUC 0.734 vs. 0.715 for sPAP, 0.615 for TAPSE). This establishes TAPSE/sPAP as a promising non‐invasive marker for CKD risk stratification [13], capturing subclinical hemodynamic changes preceding overt dysfunction [14]. Rather than reflecting renal disease progression per se, TAPSE/sPAP should be interpreted as a marker of the cardiac and hemodynamic consequences of CKD, particularly evolving RV–PA uncoupling.

Age strongly associated with CKD stage, consistent with age‐related renal decline [1]. Unlike healthy populations where TAPSE/sPAP remains stable with aging [15], our CKD cohort showed significant age‐related deterioration (*r* = –0.187, *p* = 0.041) with concurrent sPAP elevation (*r* = 0.219, *p* = 0.016). Random forest analysis (AUC 0.861 ± 0.063) identified age as the dominant predictor (35.8% importance), followed by TAPSE/sPAP (22.0%)—substantially outperforming its components (sPAP 12.6%, TAPSE 8.4%). This underscores aging's profound influence on CKD pathophysiology and confirms TAPSE/sPAP superiority as an integrated metric capturing both RV function and afterload. Epidemiological data showing 40%–50% of individuals >75 years having stage III+ CKD [16] reflects the intertwined nature of physiological aging and pathological renal decline.

Age‐stratified analyses suggested that the discriminative performance of TAPSE/sPAP for identifying advanced CKD categories was more pronounced in patients younger than 65 years than in older adults (<65 year: AUC 0.819, cutoff 0.19 vs. ≥65 year: AUC 0.579, cutoff 0.69; *p* = 0.015). This reflects narrower TAPSE/sPAP separation across CKD stages in older adults (0.583 ± 0.123 vs. 0.545 ± 0.131, *p* = 0.215) compared to younger patients (0.648 ± 0.119 vs. 0.497 ± 0.118, *p* < 0.001), likely due to age‐driven baseline sPAP elevation and higher advanced CKD prevalence (68.4% vs. 17.1%). While prior studies demonstrated TAPSE/sPAP prognostic value in elderly heart failure [17] and systemic sclerosis [18], this is the first systematic evaluation of age‐specific discriminatory performance and optimal cutoffs. Our findings suggest age‐adjusted interpretation or complementary biomarkers may be necessary for older CKD patients, consistent with observations that aging modulates structural cardiac parameter‐outcome relationships [19, 20].

LVEF significance in the fully adjusted component model requires cautious interpretation: strong age correlation (*r *= –0.631, *p* < 0.001), loss of significance in age‐excluded models, and directional change indicate an age‐related suppressor phenomenon rather than independent causality, underscoring the complex aging‐cardiac‐renal interplay.

### 4.1 Mechanistic Considerations: From Renal Dysfunction to RV‐PA Uncoupling

Progressive RV‐PA uncoupling in CKD reflects converging mechanisms: volume overload elevates pulmonary pressures via left atrial hypertension and reactive vasoconstriction [21]; uremic milieu promotes vascular remodeling with reduced NO bioavailability [22]; FGF‐23 induces myocardial fibrosis impairing RV contractility [23]; anemia increases cardiac demands; and RAAS activation causes hypertension and myocardial apoptosis [24]. This pattern is consistent with the pathophysiological framework of type 4 cardiorenal syndrome (renocardiac syndrome), in which chronic kidney disease contributes to secondary cardiac structural and functional impairment. These create progressive RV‐PA uncoupling wherein RV contractile reserve becomes insufficient for rising afterload—captured by declining TAPSE/sPAP [14, 25].

CKD‐related neurohormonal activation (RAAS upregulation [26], FGF‐23 elevation [23]) may directly contribute to RV remodeling independent of pulmonary vascular disease. Cardiac MRI studies report larger RV volumes and impaired strain in advanced CKD despite preserved LV function. Studies in hemodialysis populations show 30%–50% pulmonary hypertension prevalence [22, 27], with fluid overload as a modifiable contributor. The pathophysiological cascade involves chronic volume expansion leading to increased pulmonary blood flow, capillary stress failure, and progressive vascular remodeling [21]. Studies in pulmonary hypertension have demonstrated that right heart size, including right atrial and ventricular areas, correlates with RV‐PA coupling indices such as the TAPSE/sPAP ratio [22, 27], suggesting that progressive cardiac chamber remodeling reflects cumulative hemodynamic burden. Advanced imaging documents subclinical RV remodeling and impaired mechanics in advanced CKD despite preserved LV function [28].

The TAPSE/sPAP ratio demonstrates robust RV‐PA coupling assessment across heart failure (38), pulmonary hypertension [13, 25, 29] and CKD. In our multivariate analyses, individual TAPSE and sPAP contributions lost significance when TAPSE/sPAP was added, confirming it as an independent holistic marker with highest diagnostic accuracy (AUC 0.734). The TAPSE/sPAP ratio demonstrates robust RV‐PA coupling assessment across heart failure (38), pulmonary hypertension and CKD. In our multivariate analyses, individual TAPSE and sPAP contributions lost significance when TAPSE/sPAP was added, confirming it as an independent holistic marker with highest diagnostic accuracy (AUC 0.734). DeLong pairwise comparisons provided important nuance: TAPSE/sPAP was the only parameter to significantly outperform TAPSE alone (*Z* = 1.967, *p* = 0.049), while its discriminatory performance was statistically comparable to sPAP (*Z* = 0.843, *p* = 0.400). This statistical equivalence, however, does not diminish the clinical value of the integrated ratio. Unlike sPAP alone—which reflects only the hemodynamic burden imposed on the RV—TAPSE/sPAP simultaneously captures RV contractile adaptation to afterload, providing a physiologically richer assessment. In clinical practice, two patients with identical sPAP values may have markedly different RV functional reserves, a distinction that sPAP alone cannot capture but TAPSE/sPAP readily quantifies—a distinction particularly relevant in CKD where progressive RV‐PA uncoupling may develop insidiously before overt decompensation.

TAPSE/sPAP associates with cardiovascular events, intradialytic hypotension, eGFR, and mortality [18, 27]. Our study found 62.5% sensitivity/83.7% specificity at ≤0.52 cutoff for stage III–IV CKD. For screening, a sensitivity‐oriented threshold (≤0.595; 72.5% sensitivity/65.0% specificity) may reduce missed cases, retaining ≤0.52 as a specific high‐likelihood threshold. Remarkably, the 0.55 cutoff used in pulmonary hypertension diagnosis [9] aligns with systemic sclerosis‐CKD findings [18], suggesting this represents a universal RV‐PA coupling compromise threshold reflecting shared RV‐afterload mismatch pathophysiology [13, 25]. Our low‐pulmonary‐hypertension‐risk cohort (excluding PTE, heart failure, COPD, OSAS, PAH, stage V CKD, dialysis) suggests TAPSE/sPAP relates closely to renal failure beyond pulmonary hemodynamics. While lacking mortality follow‐up, lower ratios (≤0.32, ≤0.19 mm/mmHg) worsen prognosis in pulmonary hypertension and CKD (12, 23), supporting TAPSE/sPAP utility in both CKD staging and long‐term cardiovascular risk prediction.

### 4.2 Clinical Perspectives

**What is known**: The TAPSE/sPAP ratio is an established marker of right ventricular–pulmonary artery coupling and predicts outcomes in heart failure and pulmonary hypertension. Chronic kidney disease (CKD) is associated with increased cardiovascular morbidity through bidirectional cardiorenal interactions, yet the age‐specific performance of TAPSE/sPAP in CKD populations remains unexplored, limiting its clinical utility for risk stratification across diverse age groups.

**What this study adds**: In age‐stratified analyses, TAPSE/sPAP showed greater discriminative performance for identifying advanced CKD categories in younger patients than in older patients within this cohort. Random forest analysis with 5‐fold cross‐validation confirmed age as the dominant predictor (35.8% feature importance) and TAPSE/sPAP as the second most important variable (22.0%), outperforming individual TAPSE or sPAP measurements. Adjusted models demonstrated that each 0.10‐unit decrease in TAPSE/sPAP was independently associated with higher odds of being in a more advanced CKD category (OR 2.08, 95% CI 1.49–2.89, *p* < 0.001), highlighting the marker's clinical relevance for detecting RV‐PA uncoupling in cardiorenal syndrome.

**What are the clinical implications**: Age‐stratified TAPSE/sPAP interpretation enables more precise cardiovascular risk assessment during routine echocardiography in CKD patients. For patients <65 years, a cutoff of ≤0.19 mm/mmHg (sensitivity 78.6%, specificity 86.8%) reliably identifies those at risk for progressive renal dysfunction who may benefit from earlier cardiology referral, optimization of volume status, and RAAS blockade intensification. In contrast, for patients ≥65 years, the limited discriminatory capacity (AUC 0.579) necessitates integration with established renal biomarkers—such as albuminuria and eGFR trajectory—rather than isolated use. Serial TAPSE/sPAP monitoring may guide nephrology referral timing and medical optimization in younger CKD cohorts, while future studies should explore whether longitudinal changes—rather than single measurements—improve prognostic accuracy in older adults and whether combining TAPSE/sPAP with novel biomarkers (NGAL, KIM‐1) enhances risk stratification across all ages. Adoption of age‐adjusted reference ranges is essential, given the disparate optimal cutoffs identified in this study.

## 5 Limitations

Patients with stage V CKD receiving hemodialysis were excluded primarily for measurement‐quality reasons: the TAPSE/sPAP ratio is sensitive to loading conditions, and dialysis‐related volume shift cause meaningful pre‐/post‐dialysis variability, increasing misclassification when a single echocardiographic time‐point is used. Therefore, our findings are applicable to CKD stages I–IV and may not generalizable to dialysis‐dependent stage V populations [27, 30].

Several additional limitations exist. First, this single‐center study with modest sample size, which may limit statistical power and generalizability. In addition, the relatively small sample size and the age imbalance between groups, particularly the younger control group compared with the advanced CKD group, may have influenced the observed associations and limited interpretability despite multivariable adjustment and age‐stratified analyses. Accordingly, these findings should be interpreted cautiously and confirmed in larger, prospectively designed cohorts with more age‐balanced group distributions. Second, the cross‐sectional design precludes causal inference and precludes evaluation of temporal changes in TAPSE/sPAP with CKD progression. Third, residual confounding cannot be fully excluded despite multivariable adjustment. Although patients with overt heart failure and LVEF <50% were excluded, heart failure phenotype was not formally characterized, and no specific inference regarding HFpEF can be made from the present data.

## 6 Future Directions

Future research should prioritize prospective, longitudinal studies to characterize TAPSE/sPAP trajectories as renal function declines and to determine whether early changes predict clinical outcomes (mortality, cardiovascular events, dialysis initiation). Such designs would allow standardized echocardiography timed to volume status and dialysis sessions, improving measurement reliability and enabling stage‐specific risk thresholds. Additionally, combining TAPSE/sPAP with novel renal biomarkers (NGAL, KIM‐1) and exploring its prognostic value in stage V CKD warrant investigation.

## 7 Conclusion

This study demonstrates that the TAPSE/sPAP ratio is an effective independent marker of CKD stage severity. It enables non‐invasive identification of subclinical cardiac dysfunction and pulmonary hypertension in CKD patients, with excellent discriminatory performance in younger individuals (<65 years) but limited utility in older populations. Future studies with larger samples and long‐term follow‐up are needed to validate its prognostic value and established age specific reference ranges for clinical implantation.

## Funding

This research did not receive any specific grant from funding agencies in the public, commercial, or not‐for‐profit sectors.

## Ethics Statement

This study was conducted in accordance with the principles of the Declaration of Helsinki. Ethical approval for this study was granted by the Health Sciences Research Ethics Committee of Yüksek İhtisas University (Approval Number: 2024/05/18, Date: July 31 2024).

## Conflicts of Interest

The authors certify that they have no affiliations with or involvement in any organization or entity with any financial interest (such as honoraria; educational grants; participation in speakers’ bureaus; membership, employment, consultancies, stock ownership, or other equity interest; and expert testimony or patent‐licensing arrangements) or nonfinancial interest (such as personal or professional relationships, affiliations, knowledge, or beliefs) in the subject matter or materials discussed in this manuscript.
